# oriTDB: a database of the origin-of-transfer regions of bacterial mobile genetic elements

**DOI:** 10.1093/nar/gkae869

**Published:** 2024-10-07

**Authors:** Guitian Liu, Xiaobin Li, Jiahao Guan, Cui Tai, Yuqing Weng, Xiaohua Chen, Hong- Yu Ou

**Affiliations:** Department of Infectious Diseases, Shanghai Sixth People's Hospital Affiliated to Shanghai Jiao Tong University School of Medicine, Shanghai 200233, China; State Key Laboratory of Microbial Metabolism, Joint International Laboratory on Metabolic & Developmental Sciences, School of Life Sciences & Biotechnology, Shanghai Jiao Tong University, Shanghai 200240, China; Guangdong Provincial Key Laboratory of Tumor Interventional Diagnosis and Treatment, Zhuhai People's Hospital (Zhuhai Clinical Medical College of Jinan University), Zhuhai 519000, China; State Key Laboratory of Microbial Metabolism, Joint International Laboratory on Metabolic & Developmental Sciences, School of Life Sciences & Biotechnology, Shanghai Jiao Tong University, Shanghai 200240, China; State Key Laboratory of Microbial Metabolism, Joint International Laboratory on Metabolic & Developmental Sciences, School of Life Sciences & Biotechnology, Shanghai Jiao Tong University, Shanghai 200240, China; Department of Pulmonary and Critical Care Medicine, Zhuhai People's Hospital (Zhuhai Clinical Medical College of Jinan University), Zhuhai 519000, China; Department of Infectious Diseases, Shanghai Sixth People's Hospital Affiliated to Shanghai Jiao Tong University School of Medicine, Shanghai 200233, China; Department of Infectious Diseases, Shanghai Sixth People's Hospital Affiliated to Shanghai Jiao Tong University School of Medicine, Shanghai 200233, China; State Key Laboratory of Microbial Metabolism, Joint International Laboratory on Metabolic & Developmental Sciences, School of Life Sciences & Biotechnology, Shanghai Jiao Tong University, Shanghai 200240, China

## Abstract

Conjugation and mobilization are two important pathways of horizontal transfer of bacterial mobile genetic elements (MGEs). The origin-of-transfer (*oriT*) region is crucial for this process, serving as a recognition site for relaxase and containing the DNA nicking site (*nic* site), which initiates the conjugation or mobilization. Here, we present a database of the origin-of-transfer regions of bacterial MGEs, oriTDB (https://bioinfo-mml.sjtu.edu.cn/oriTDB2/). Incorporating data from text mining and genome analysis, oriTDB comprises 122 experimentally validated and 22 927 predicted *oriT*s within bacterial plasmids, Integrative and Conjugative Elements, and Integrative and Mobilizable Elements. Additionally, oriTDB includes details about associated relaxases, auxiliary proteins, type IV coupling proteins, and a gene cluster encoding the type IV secretion system. The database also provides predicted secondary structures of *oriT* sequences, dissects *oriT* regions into pairs of inverted repeats, *nic* sites, and their flanking conserved sequences, and offers an interactive visual representation. Furthermore, oriTDB includes an enhanced *oriT* prediction pipeline, oriTfinder2, which integrates a functional annotation module for cargo genes in bacterial MGEs. This resource is intended to support research on bacterial conjugative or mobilizable elements and promote an understanding of their cargo gene functions.

## Graphical Abstract

**Graphical Abstract:**
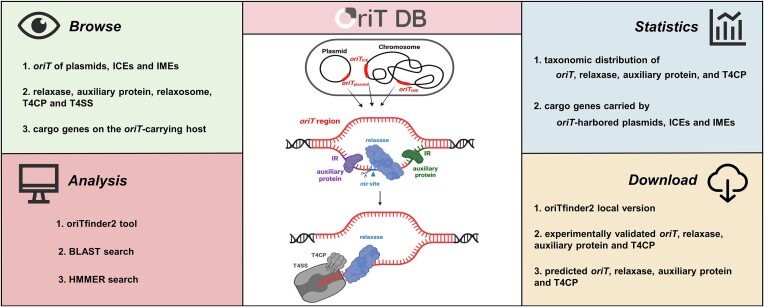

## Introduction

Bacterial mobile genetic elements (MGEs) are crucial in disseminating virulence factor genes and antimicrobial resistance genes(1). These elements, including conjugative or mobilizable plasmids, integrative and conjugative elements (ICEs), and Integrative and Mobilizable Elements (IMEs), typically consist of four modules in their conjugative regions: the origin-of-transfer (*oriT*) region, relaxase gene, type IV coupling protein (T4CP) gene, and a gene cluster encoding type IV secretion system (T4SS). The *oriT* region, usually ranging in length from tens to hundreds of base pairs, contains a *nic* site and pairs of inverted repeats (IRs) (2). It is vital for the transfer process as it is recognized by the relaxase and undergoes nicking at a conserved site (*nic*), resulting in the formation of single-stranded DNA (ssDNA) (3). This ssDNA, along with the relaxase and auxiliary proteins, forms a relaxosome and is transferred with the assistance of T4SS and its T4CPs. Therefore, it is essential to curate data on the *oriT* regions and their corresponding relaxase genes, auxiliary protein genes, T4CP genes, and the T4SS gene cluster, which help investigate the self-transfer or mobilization of bacterial MGEs.

Many studies have been conducted to investigate the potential mobility of plasmids with a focus on relaxases (4,5), by using experimental methods (6,7) and *in silico* approaches (8). Computational tools have also been developed to predict the *oriT* regions, which helps examine the transfer of MGEs. The prediction tool oriTfinder, developed by our group, employs similarity searches for *oriT* sequences and the co-localization of flanking relaxase homologous genes (9). Furthermore, a novel approach has utilized intergenic positioning, relaxase distance and MOB-type association to detect *oriT*s in plasmids of specific bacterial species (10), such as *Escherichia coli, Klebsiella pneumoniae* and *Acinetobacter baumannii*. Recently, it was discovered that plasmids lacking a relaxase retain the capability to transfer (11–13), indicating the necessity to predict plasmid mobility independent of relaxase information. Certain structural conservation has been observed in *oriT* sequences (14), allowing non-conjugative plasmids to become mobilizable by carrying *oriT*-mimics (15,16), which suggests a need for further *in silico* dissection of the *oriT* regions. The IRs and some specific regions in the *oriT* sequence serve as the binding site for the relaxase and auxiliary proteins, such as the IR2 within *oriT*_ICE_*_st3_* for the relaxase RelSt3 (17) and the *ossA* and *ossB* region within *oriT*_pWBG749_ for the auxiliary protein SmpO (16). However, existing bioinformatics resources lack comprehensive data on the *oriT* regions, including their essential *nic* sites, IRs, and the corresponding relaxases, auxiliary proteins, relaxosomes and T4CPs. Moreover, analytical tools connecting the mobility of different MGEs with the diverse cargo genes they carry are insufficient, hindering the understanding of the relationship between MGEs and their cargo genes.

Here, we present the release of oriTDB, a comprehensive database containing an expanded collection of curated *oriT* regions. Within this database are details about the IRs and *nic* sites located within the *oriT* regions, along with information about their corresponding relaxases, auxiliary proteins, and T4CPs. Additionally, a tool capable of identifying the *oriT* region in a MGE lacking a relaxase through sequence comparison against oriTDB. Moreover, oriTfinder2 integrates a novel functional annotation module for cargo genes in *oriT*-carrying MGEs. We anticipate that oriTDB will streamline the precise localization of *oriT* regions and the functional assessment of diverse cargo genes within *oriT*-carrying MGEs.

## Materials and methods

### Data updated by text mining and manual curations

Through a meticulous manual curation of PubMed literature using ‘*oriT*’ and ‘origin of transfer’ keywords, we have amassed a collection of 741 papers (on 25 March 2024). After text mining and manual curations, oriTDB collected 122 experimentally validated *oriT*s from various MGEs of 45 species, including those in plasmids (*n*= 91), ICEs (*n*= 18) and IMEs (*n*= 13). Additionally, oriTDB now incorporates information on *oriT*-related and experimentally validated relaxases (*n* = 50), auxiliary proteins (*n*= 44) and T4CPs (*n*= 16) (Supplementary Table S1). We have also compiled 21 entries for relaxosomes, providing details on the *oriT* regions, associated relaxases and auxiliary proteins.

### Prediction of *oriT* region and cargo genes in MGE sequences using oriTfinder2

The updated tool, oriTfinder2, aims to predict potential *oriT*s, relaxases and T4CPs in plasmids, ICEs, and IMEs (Supplementary Figure S1). It has been enhanced to improve the prediction accuracy for relaxases by utilizing hidden Markov model (HMM) profiles for the MOB protein family (Supplementary Table S2). Additionally, oriTfinder2 can identify *oriT* regions in a relaxase-lacking mobilizable plasmid by integrating BLASTn searches against the *oriT* sequences archived in the oriTDB (Supplementary Method and Supplementary Figure S1). The plasmid carrying the *oriT* but lacking a relaxase gene was found to be mobilized with the help of a conjugative plasmid(13). By utilizing oriTfinder2, we have identified 22 927 putative *oriT* regions (22 390 in plasmids, 482 in ICEs, and 55 in IMEs) across 972 species from MGE sequences sourced from the NCBI RefSeq (18) plasmid dataset (*n*= 86 009) (on 21 March 2024) and our ICE database ICEberg 3.0 (1323 ICEs and 324 IMEs) (Supplementary Table S1). The number and ratio of *oriT*-carrying plasmid, ICEs and IMEs predicted by oriTfinder 2.0 are 22 199 (25.8%), 457 (34.5%) and 68 (20.9%), respectively. These pre-computing results were stored by oriTDB.

Furthermore, oriTfinder2 also incorporates a functional annotation pipeline for cargo genes in *oriT*-carrying MGEs, encompassing acquired antibiotic resistance genes sourced from Resfinder (19), virulence factors obtained from VFDB (20), metal resistance determinants from BacMet2 (21), anti-CRISPR proteins from Anti-CRISPRdb v2.2 (22), microbial degradation proteins from mibPOPdb (23) and symbiotic proteins compiled by ICEberg 3.0 (24). More details are available in the Supplementary Methods and Supplementary Table S3. This advancement may contribute to a comprehensive understanding of various biological functionalities associated with bacterial MGEs.

### Implementation of the web-based database oriTDB

The oriTDB utilizes a PostgreSQL relational database, a PHP data pipeline, and HTML web interfaces for data management. It also incorporates the Bootstrap library (https://getbootstrap.com/) and JavaScript-powered data visualization libraries, such as ECharts (25) and SVGene (https://github.com/kblin/svgene), to improve user interaction. Sequence feature annotations of *oriT*, including IRs and conserved nick regions, are visualized using seqviz (https://github.com/Lattice-Automation/seqviz). Additionally, DNA secondary structures of *oriT* regions were predicted by RNAfold with the DNA parameters (26). The circular genome visualization tool CGView (27) is integrated into the oriTfinder2 result page to depict the distribution of the putative *oriT* and other transfer modules in the MGE sequence. Moreover, the Pfam (28) domains of the proteins related to *oriT* are predicted using InterProScan(29). Experimentally determined and predicted 3D structures for each protein are obtained from the RCSB Protein Data Bank (PDB) (https://www.rcsb.org/)(30) and the AlphaFold Protein Structure Database (31), respectively, and are interactively visualized using PDBe Mol* (32). Finally, DNA and protein homologs are identified using NCBI BLASTp (33) and HMMER3 (34) in the BLASTp and HMMER search tools.

## Results and discussion

### Compilation and visualization of *oriT* regions

Through a rigorous process of text mining and manual curation, we have introduced 23 527 *oriT* entries, 13 116 relaxase entries, 7658 auxiliary protein entries, 21 relaxosome entries and 16 985 T4CP entries into oriTDB, making it a robust and comprehensive database for *oriT* and its related elements. Notably, a substantial subset of 122 *oriT*s, 50 relaxases, 44 auxiliary proteins, 6 relaxosomes and 16 T4CPs has been experimentally validated, emphasizing the reliability of the database content (Supplementary Table S1). Additionally, our analysis has documented six categories of cargo genes found on *oriT*-carrying MGEs, encompassing acquired antimicrobial resistance genes, virulence factor genes, metal resistance genes, degradation genes, symbiosis genes, and anti-CRISPR genes. This information holds potential for enriching the understanding of the network involving *oriT*-MGEs-cargo genes across diverse bacterial species.

**Figure 1.**
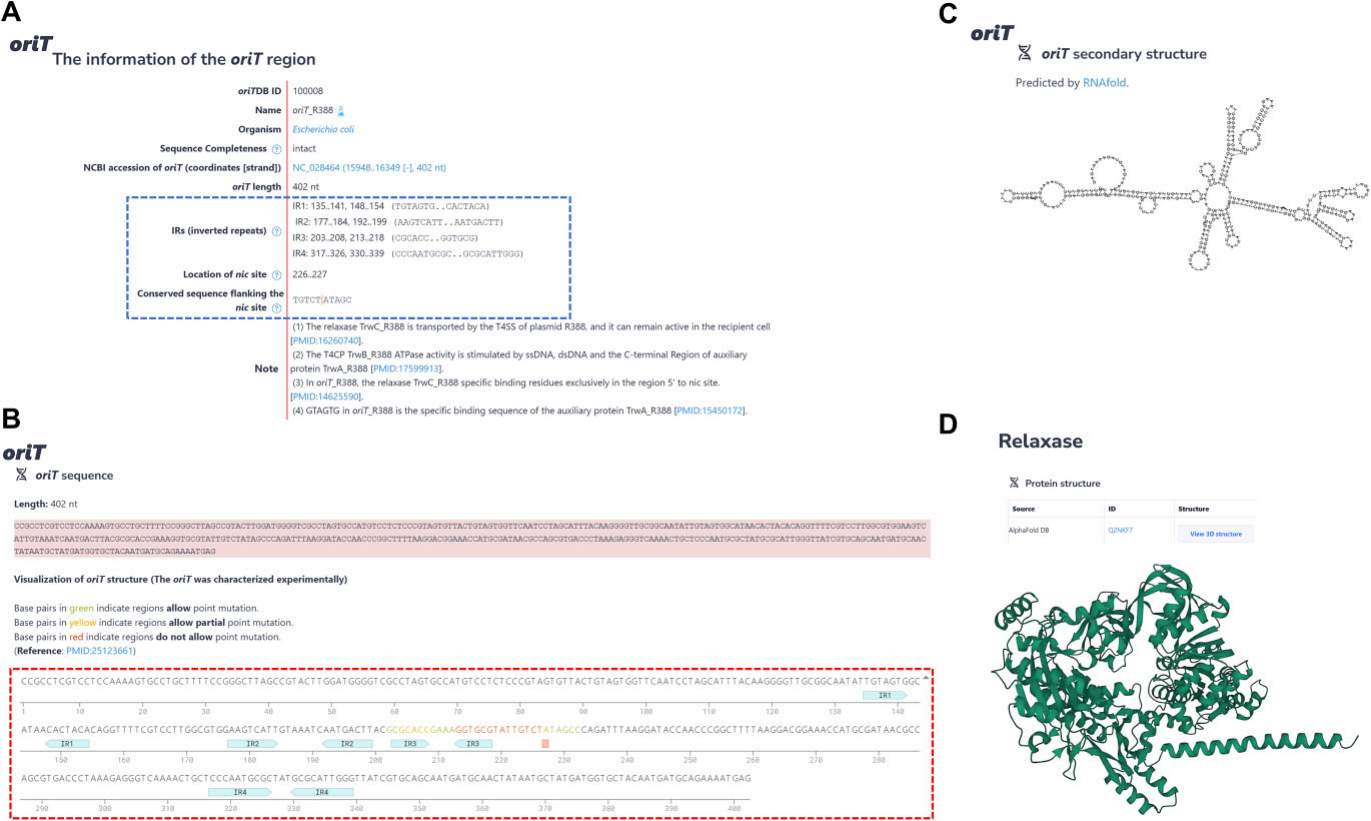
Overview of the dissection and visualization of the *oriT* region in oriTDB. (**A**) The detailed information of the *oriT*_R388 region in the plasmid R388 of *Escherichia coli*. The *oriT* region was dissected into pairs of IRs, *nic* sites, and their flanking conserved sequences (blue dashed box). (**B**) Visual representation of the *oriT*_R388 region. The sequence of *oriT* is presented in the interactive visualization form (red dashed box), with the IR pairs in the *oriT* region as aqua arrows, and the *nic* sites as orange rectangles. (**C**) The RNAfold-predicted secondary structure of the single strand *oriT*_R388 region. (**D**) The AlphaFold2-predicted structure of the relaxase protein TrwC_R388 related to *oriT*_R388.

To further study in *oriT* region, we have dissected and provided interactive visualization of the compiled *oriT* in oriTDB (Figure 1). In the *oriT* entries, comprehensive information about *oriT*s, including the IRs, *nic* sites, conserved flanking sequences, regular sequences, and other specialized regions was provided (Figure 1A). Detailed information about *oriT* regions is visually presented in oriTDB, especially for the experimentally validated ones (Figure 1B). Furthermore, for the recorded *oriT* sequences, oriTDB employed RNAfold to predict and display their secondary structures, which display the complex relationships between the dissected elements within the *oriT* region through user-friendly web pages (Figure 1C) (21). Additionally, oriTDB provides information on the *oriT-*related relaxase (Figure 1D), auxiliary protein, T4CP and T4SS. Together, based on the dissection and interactive visualization of the *oriT* region, oriTDB provides a deeper understanding of the dissected elements within the *oriT* region.

### Categorization for cargo genes in *oriT*-carrying MGEs

**Figure 2.**
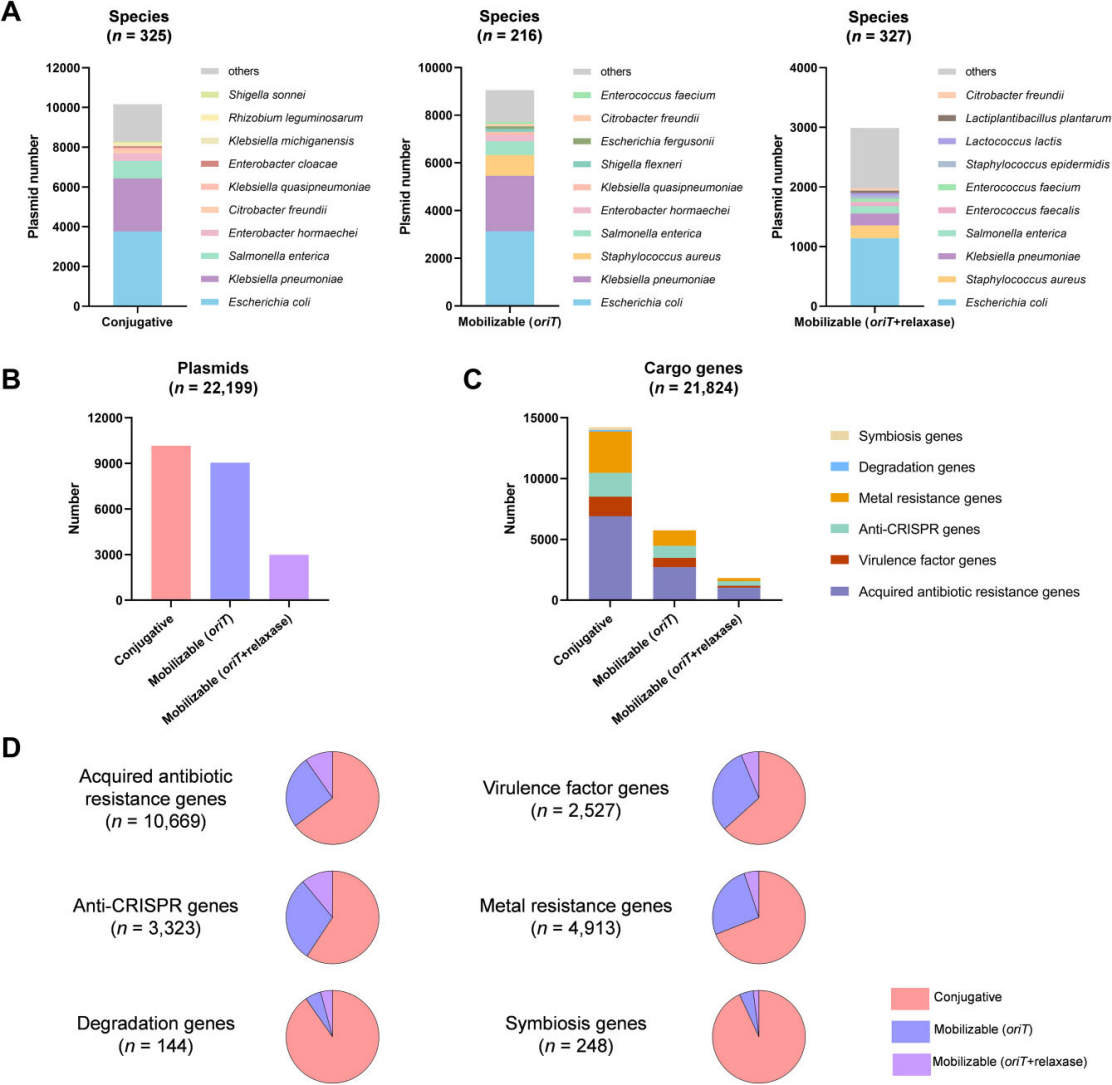
Species distribution and cargo genes categorization of the *oriT*-carrying plasmids predicted by oriTfinder2. (**A**) Species distribution of the *oriT*-carrying plasmids. (**B**) The number of the conjugative, mobilizable (*oriT*) and mobilizable (*oriT*+ relaxase) plasmids. (**C**) The number of the six categorized cargo genes in the *oriT*-carrying plasmids. (**D**) The proportion of the three categorized *oriT*-carrying plasmids in the six categorized cargo genes.

Bacterial MGEs carry a variety of cargo genes that can provide a competitive advantage to host bacteria. However, there is limited research on the relationship between the mobility of MGEs and their cargo genes (15,35). Previous studies have primarily focused on the mobility of specific types of MGEs and their associated cargo genes (11,36–38). To address this gap, we employed the oriTDB databases to investigate the potential mobility of plasmids and their correlation with carried cargo genes. Utilizing the plasmid classification based on transfer potential described in our previous work (13), we categorized the *oriT*-carrying plasmids (*n* = 22 199) predicted by oriTfinder2 into ‘Conjugative’ (carrying the *oriT*, relaxase gene, T4CP gene, and T4SS gene cluster) (*n* = 10 164), ‘Mobilizable (*oriT*)’ (carrying the *oriT* but lacking a relaxase gene) (*n* = 9 046) and ‘Mobilizable (*oriT*+ relaxase)’ (carrying the *oriT* and a relaxase gene) (*n* = 2 989) (Figure 2B). Then we analyzed the species distribution (Figure 2A) and cargo genes associated with each category of the *oriT*-carrying plasmids (Figures 2C and D).

The 22 199 *oriT*-carrying plasmids are distributed across 583 species, such as *Escherichia coli, Klebsiella pneumoniae, Salmonella enterica, Enterobacter hormaechei, Enterococcus faecalis, Staphylococcus aureus* and *Citrobacter freundii* (Figure 2A). Of these, conjugative, mobilizable (*oriT*), and mobilizable (*oriT* + relaxase) plasmids are found in 325, 216 and 327 species, respectively (Supplementary Table S4). Notably, about 40.7% of *oriT*-carrying plasmids do not contain the relaxase gene (Figure 2B). Recent research has shown that more than half of the plasmids in *S. aureus* are classified as ‘Mobilizable (*oriT*)’(11,15). Our previous conjugation experiments have observed that mobilizable plasmids lacking the relaxase gene were transferable in *K. pneumoniae* through interaction with a helper conjugative plasmid (Supplementary Figure S2) (12,13). These findings imply that a considerable proportion of ‘Mobilizable (*oriT*)’ plasmids possess transferability. Additionally, the species of *oriT*-carrying ICEs and IMEs are detailed in Supplementary Figure S3 and Supplementary Table S5.

The primary focus of oriTDB has been to systematically classify and integrate cargo gene functions associated with *oriT*-carrying MGEs. The ‘Browse’ web page of oriTDB presents categorized cargo gene function lists for individual MGEs, allowing users to access detailed information about the cargo genes conveniently. Through *in silico* analysis using oriTfinder2, prevalent cargo genes within plasmids, ICEs, and IMEs have been categorized into six distinct groups (Figure 2C). Among 22 199 *oriT*-carrying plasmids, 21 824 cargo genes were annotated (Figure 2C). Furthermore, 14 233, 5761 and 1830 cargo genes were found in conjugative plasmids, mobilizable (*oriT*) plasmids, and mobilizable (*oriT*+ relaxase) plasmids, respectively (Supplementary Table S4). Additionally, the cargo genes of *oriT*-carrying ICEs and IMEs are presented in Supplementary Figure S4 and Supplementary Table S5. Utilizing the oriTDB to analyze the cargo genes carried by MGEs may assist researchers in exploring the correlation between the potential mobility of the MGEs and their cargo genes, thereby providing further insights into the realm of MGE biology.

## Conclusion

In this report, we introduced a user-friendly *oriT* database, accompanied by an enhanced *oriT* prediction tool. The oriTDB provides a comprehensive compilation of both experimentally validated and predicted *oriT* regions, along with their associated relaxases, auxiliary proteins, and T4CPs in bacterial plasmids, ICEs and IMEs. Additionally, oriTDB offers comprehensive information on the IRs and *nic* sites in the *oriT* region to support experimental research. Furthermore, the upgraded oriTfinder2 has not only improved the accuracy of its *oriT* region predictions but also incorporates annotation features for cargo genes in MGEs. The oriTDB database will be continuously maintained and updated to align with the rapidly expanding microbial genome database and ensure its ongoing relevance.

## Supplementary Material

gkae869_Supplemental_Files

## Data Availability

oriTDB is freely available at https://bioinfo-mml.sjtu.edu.cn/oriTDB2/ .
