# Supplementary material for: oriTDB: a database of the origin-of-transfer regions of bacterial mobile genetic elements
Source: Nucleic Acids Res. 2024 Oct 7;53(D1):D163–8. doi: 10.1093/nar/gkae869 (PMC11701681; doi:10.1093/nar/gkae869)
Supplement: gkae869_Supplemental_Files [file gkae869_supplemental_files.zip › Supplementary_file.pdf]

oriTDB: a database of the origin-of-transfer regions of bacterial mobile genetic elements

SUPPLEMENTARY METHODS

oriTfinder2 predicting *oriT*s and cargo genes in bacterial MGE sequences

SUPPLEMENTARY DATA

Table S1. Statistical summary of *oriT*s, relaxases, auxiliary proteins, T4CPs and relaxosomes archived in oriTDB.

Table S2. Profile Hidden Markov Models (HMM) for relaxases and T4CPs used by oriTfinder2.

Table S3. The prediction strategy of detecting cargo genes in a bacterial plasmid, ICE, or IME used by oriTfinder2.

Table S4. Species distribution and categorized cargo genes of the conjugative, mobilizable (*oriT*) and mobilizable (*oriT*+relaxase) plasmids.

Table S5. Species distribution and categorized cargo genes of the *oriT*-carrying ICEs and IMEs.

Figure S1. The prediction strategy used by oriTfinder2 to identify the *oriT region*, relaxase gene, T4CP gene, T4SS gene cluster, and cargo genes in a DNA sequence of bacterial plasmid, ICE, or IME.

Figure S2. Overview of oriTfinder2 outputs with two examples of the *oriT*-carrying plasmid and the mobilizable (*oriT*) plasmid.

Figure S3. Species distribution of the *oriT*-carrying ICEs and IMEs.

Figure S4. The number of the categorized cargo genes in the *oriT*-carrying ICEs and IMEs.

SUPPLEMENTARY METHODS

oriTfinder2 predicting *oriT*s and cargo genes in bacterial MGE sequences

The oriTfinder2 has been updated to improve *oriT* detection capabilities by expanding the experimental dataset from 52 to 122 *oriT* sequences in the background dataset oriTDB. It can now identify nine relaxase families and three T4CP families through an increased number of HMM profiles (Supplementary Table S2). Additionally, the tool can detect T4SS by integrating the CONJScan model from the macsyfinder software(1).

In addition to *oriT* detection, oriTfinder2 includes a functional annotation module for identifying cargo genes in bacterial MGEs. We established a comprehensive dataset containing acquired antibiotic resistance genes sourced from Resfinder(2), virulence factors obtained from VFDB(3), anti-CRISPR proteins from Anti-CRISPRdb v2.2(4), metal resistance determinants from BacMet2(5), microbial degradation proteins from mibPOPdb(6), and symbiotic proteins compiled by ICEberg3.0(7). Specifically, the symbiotic proteins are focused on the nitrogen-fixing symbiosis of rhizobia. The identification of cargo genes is carried out through BLAST searches using specified parameters (Supplementary Table S3).

Table S1. Statistical summary of *oriT*s, relaxases, auxiliary proteins, T4CPs and relaxosomes archived in oriTDB.

		Number of elements derived from experimental data	Number of elements derived from predicted data	Total
*oriT*DB (released in 2018)				
oriT	Plasmid	42	954	996
	ICE	6	67	73
	IME	4	2	6
relaxase		27	956	983
auxiliary protein		29	73	102
T4CP		12	452	464
*oriT*DB (updated in 2024)				
oriT	Plasmid	91	22,390	22,481
	ICE	18	482	500
	IME	13	55	68
relaxase		50	13,066	13,116
auxiliary protein		44	7,614	7,658
T4CP		16	16,969	16,985
relaxosome		6	15	21

Table S2. Profile Hidden Markov Models (HMM) for relaxases and T4CPs used by oriTfinder2

**Conserved domain**	**Pfam ID** [a]	**Family**
Relaxase		
Replic_Relax	PF13814	MOB_C
TrwC	PF08751	MOB_F
TraI	PF07057	MOB_F
TraI_2	PF07514	MOB_H
Relaxase	PF03432	MOB_P
MobA_MobL	PF03389	MOB_Q
Mob_Pre	PF01076	MOB_V
Rep_trans	PF02486	MOB_T
DUF5712 [b]	PF18976	MOB_B
MobL [b]	PF18555	MOB_L
T4CP		
T4SS-DNA_transf	PF02534	t4cp1
TrwB_AAD_bind	PF10412	t4cp2
FtsK_SpoIIIE [b]	PF01580	tcpA

[a] Pfam ID, protein family database accession number (http://pfam-legacy.xfam.org/).
[b] The HMM profile newly added by oriTfinder2.

Table S3. The prediction strategy of detecting cargo genes in a bacterial plasmid, ICE, or IME used by oriTfinder2.

Cargo genes	Method	Dataset	Ref.
Antibiotic resistance genes (ARGs)	Abricate (identities≥80%, coverage≥80%)	Resfinder database	(2)
Virulence factors	BLASTp (E-value ≤0.0001; Ha-value ≥ 0.64)	VFDB database	(3,7)
Metal resistance determinants	BLASTp (E-value ≤0.0001; Ha-value ≥ 0.64)	metal resistance proteins from BacMet2	(7)
Degradation proteins	BLASTp (E-value ≤0.0001; Ha-value ≥ 0.64)	degradation proteins from mibPOPdb	(7)
Symbiosis proteins	BLASTp (E-value ≤0.0001; Ha-value ≥ 0.64)	symbiotic proteins compiled by ICEberg3	(7)
Anti-CRISPR proteins	BLASTp (E-value ≤0.001)	Anti-CRISPRdbv2.2-Verified+Pliterature	(4,8)

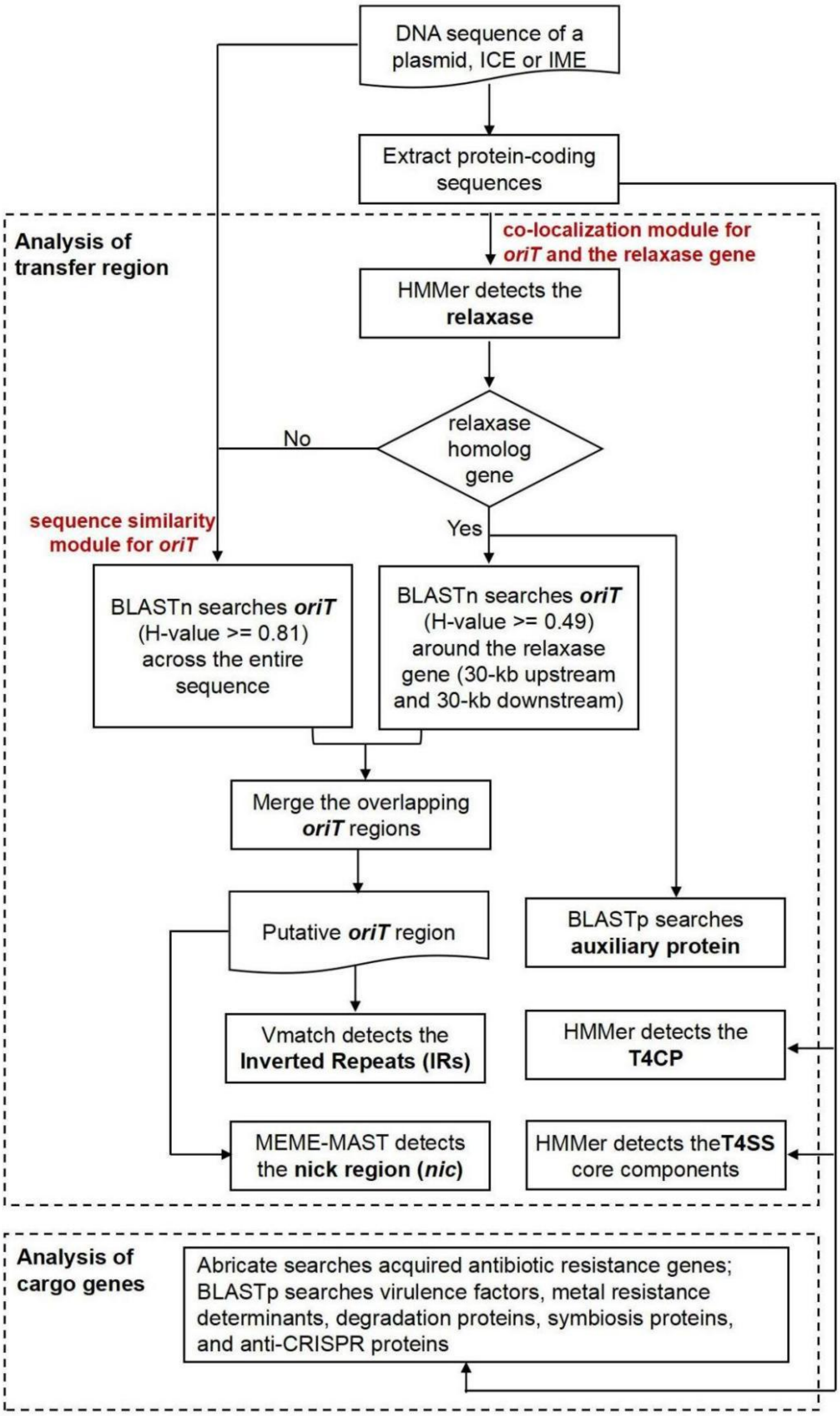

Figure S1. The prediction strategy used by oriTfinder2 to identify the *oriT region*, relaxase gene, T4CP gene, T4SS gene cluster, and cargo genes in the DNA sequence of a bacterial plasmid, ICE, or IME.

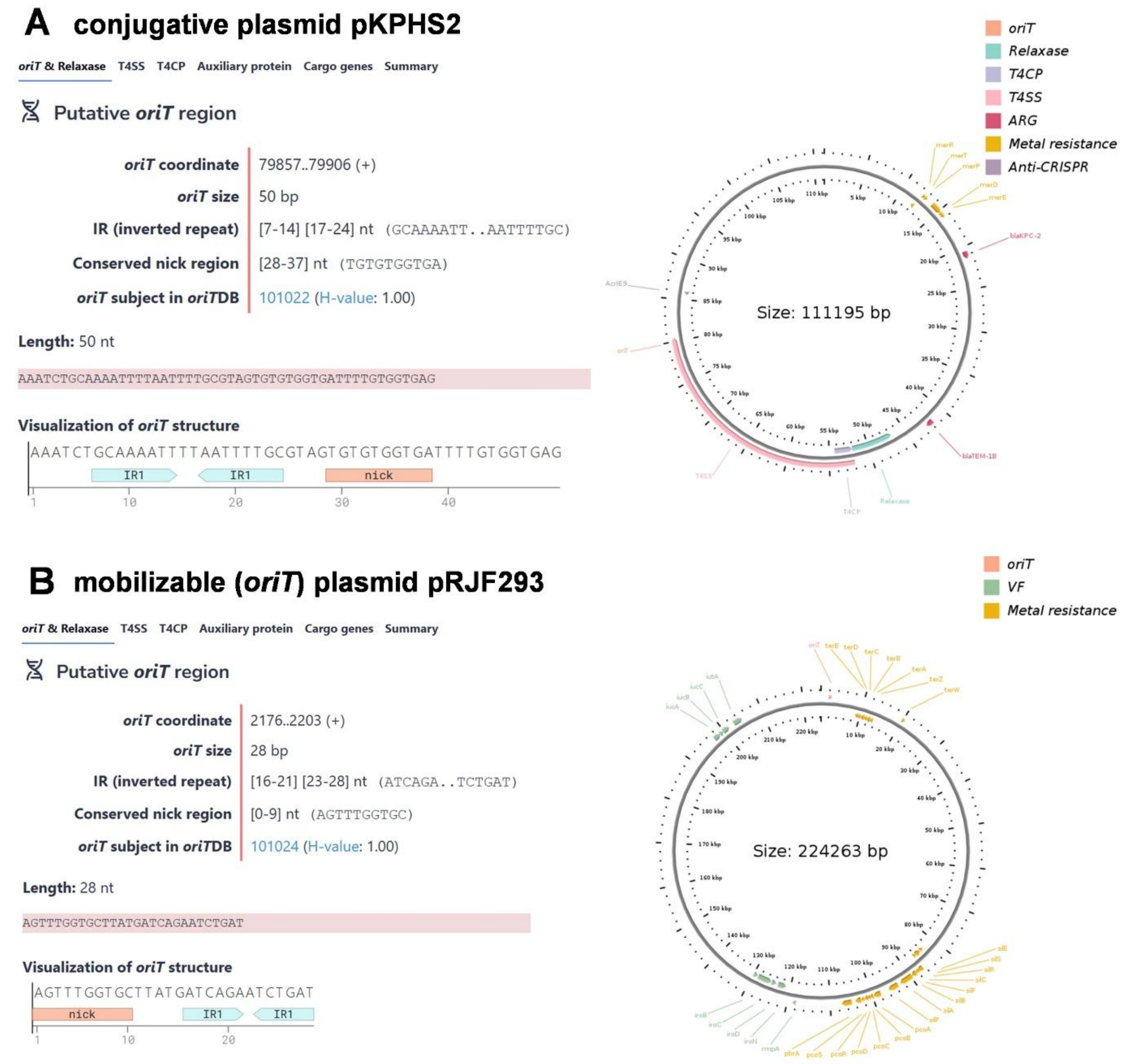

Figure S2. Overview of oriTfinder2 outputs with two examples of the *oriT*-carrying plasmid and the mobilizable (*oriT*) plasmid. (A) The conjugative plasmid pKPHS2 of *Klebsiella pneumoniae* HS11286 (NCBI accession no. NC_016846). (B) The mobilizable (*oriT*) plasmid pRJF293 of *Klebsiella pneumoniae* RJF293 (NCBI accession no. NZ_CP014009). The *oriT* region, relaxase gene, T4CP gene, gene cluster coding for T4SS, and cargo genes are marked in the circular visualization of the plasmid in different colors.

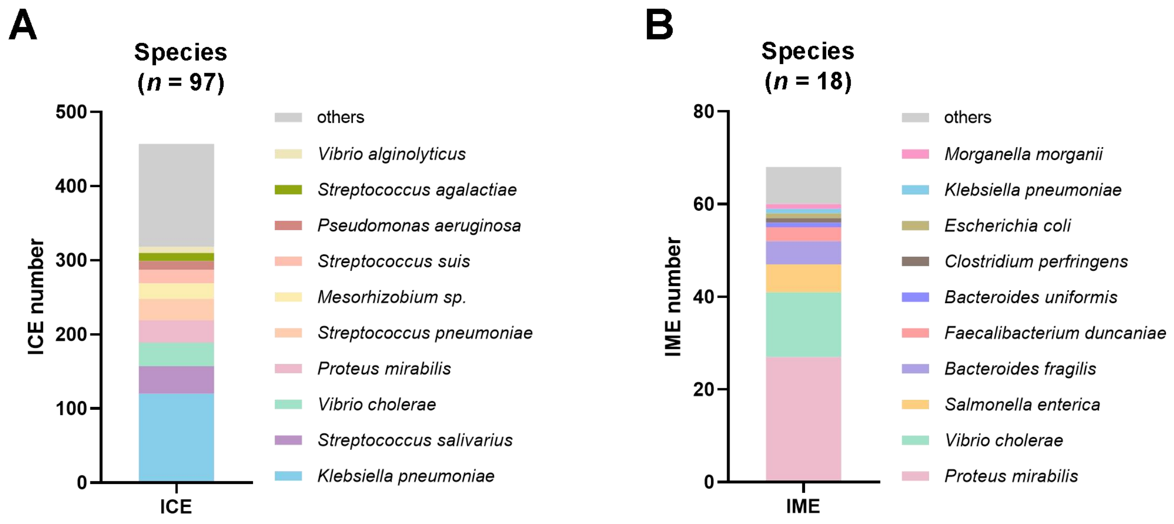

Figure S3. Species distribution of the *oriT*-carrying ICEs (A) and IMEs (B).

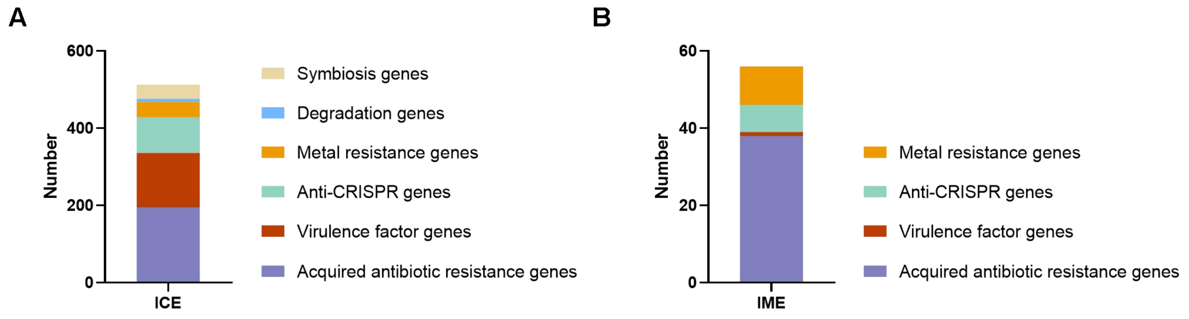

Figure S4. The number of the categorized cargo genes in the *oriT*-carrying ICEs (A) and IMEs (B).

REFERENCES

1. Abby, S.S., Néron, B., Ménager, H., Touchon, M. and Rocha, E.P.C. (2014) MacSyFinder: a program to mine genomes for molecular systems with an application to CRISPR-Cas systems. *PLoS One*, **9**, e110726.
2. Wang, M., Goh, Y.X., Tai, C., Wang, H., Deng, Z. and Ou, H.Y. (2022) VRprofile2: detection of antibiotic resistance-associated mobilome in bacterial pathogens. *Nucleic Acids Res*, **50**, W768-w773.
3. Liu, B., Zheng, D., Zhou, S., Chen, L. and Yang, J. (2022) VFDB 2022: a general classification scheme for bacterial virulence factors. *Nucleic Acids Research*, **50**, D912-D917.
4. Dong, C., Wang, X., Ma, C., Zeng, Z., Pu, D.K., Liu, S., Wu, C.S., Chen, S., Deng, Z. and Guo, F.B. (2022) Anti-CRISPRdb v2.2: an online repository of anti-CRISPR proteins including information on inhibitory mechanisms, activities and neighbors of curated anti-CRISPR proteins. *Database (Oxford)*, **2022**.
5. Pal, C., Bengtsson-Palme, J., Rensing, C., Kristiansson, E. and Larsson, D.G. (2014) BacMet: antibacterial biocide and metal resistance genes database. *Nucleic Acids Res*, **42**, D737-743.
6. Ngara, T.R., Zeng, P. and Zhang, H. (2022) mibPOPdb: An online database for microbial biodegradation of persistent organic pollutants. *Imeta*, **1**, e45.
7. Wang, M., Liu, G., Liu, M., Tai, C., Deng, Z., Song, J. and Ou, H.-Y. (2024) ICEberg 3.0: functional categorization and analysis of the integrative and conjugative elements in bacteria. *Nucleic Acids Research*, **52**, D732-D737.
8. Liu, M., Li, X., Xie, Y., Bi, D., Sun, J., Li, J., Tai, C., Deng, Z. and Ou, H.Y. (2019) ICEberg 2.0: an updated database of bacterial integrative and conjugative elements. *Nucleic acids research*, **47**, D660-d665.
